# Mechanism of Laccase Induction via Emodin in *Trametes versicolor*

**DOI:** 10.3389/fbioe.2021.653800

**Published:** 2021-05-18

**Authors:** Lin Wang, Xuecai Luo, Yu Pan, Zai Zheng, Ruochun Yin, Xiaohe Tian, Liang Zhang

**Affiliations:** ^1^School of Life Sciences, Anhui University, Hefei, China; ^2^Hefei Tingxiandu Biological Technology Co., Ltd., Hefei, China; ^3^National Engineering Laboratory for Cereal Fermentation Technology, Jiangnan University, Wuxi, China

**Keywords:** laccase, *Trametes versicolor*, emodin, proteomics analysis, CLSM

## Abstract

Secondary metabolites of traditional Chinese herbs can prominently stimulate the production of laccase from white rot fungi during submerged fermentation. However, the molecular mechanism through which these natural products induce the production of laccase remains unknown. In this study, the Chinese herbal medicine *Polygonum cuspidatum* was used to induce laccase production in *Trametes versicolor*, and the best inducer was identified in emodin, even under conditions of 1000-L, large-scale fermentation. Proteomics analysis identified a selection of proteins that were differentially expressed in the presence of emodin, indicating that emodin may affect the expression of laccase genes through three mechanisms: reducing bioenergy productivity, the aryl hydrocarbon receptor (AHR)/xenobiotic response element (XRE) pathway, and the nuclear erythroid 2-related factor 2 (Nrf2)/antioxidant response element (ARE) pathway. Combined with protoplast flow cytometry and fluorescence, it is revealed that emodin might reduce the synthesis of ATP by lowering the mitochondrial membrane potential, leading to the subsequent responses.

## Introduction

*Polygonum cuspidatum* is a common raw material used during the extraction of traditional Chinese herbs (TCH) and serves as a source of emodin, resveratrol, and other natural products. Emodin, which is the primary active substance in *P. cuspidatum*, activates blood circulation, disperses blood stasis, dredges the meridians, and has an antitussive effect (Shukla et al., 2020). The medicinal mushroom *Trametes versicolor* had been used for the microbial fermentation and transformation of TCH (Bains and Chawla, 2020). In recent years, several groups have explored the ability of *T. versicolor* to secrete laccase isozymes (Iimura et al., 2018; Pinheiro et al., 2020) and have attempted to optimize enzyme production (Yang et al., 2015; Zdarta et al., 2017), although the abilities of TCH to induce the production of laccase has not been well-explored. Laccase (benzenediol: oxygen oxidoreductase EC 1.10.3.2.) belongs to a family of blue multicopper oxidases produced by fungi, which has also been detected in plants, bacteria, and insects (Patel et al., 2018). Due to diverse substrate conformations and environmentally friendly features, laccase has been widely used for various industrial applications, such as pulp delignification and bleaching (Widsten and Kandelbauer, 2008), dye decolorization (Rangabhashiyam et al., 2013), the detoxification of environmental pollutants, and biosensors (Li et al., 2009; Mate and Alcalde, 2016). To meet the high demand for low-cost laccase, increasing laccase production is necessary. Various strategies have been developed to enhance laccase production, such as controlling the carbon-to-nitrogen ratio using inducers and lignocellulose (Bettin et al., 2009; Kuhar and Papinutti, 2014) and applying industrial and agricultural waste or by-products containing laccase inducers (Gonzalez et al., 2013). The mechanism through which these inducers act on fungal cells remains unclear because few studies have examined the process at the micro-level.

Several methods have been developed that can be applied to the in-depth exploration of how emodin accelerates the expression of laccase genes by *T. versicolor*. Mass spectrometry (MS)-based label-free quantification (LFQ) methods have previously been used for fungal proteomics examinations (Zixuan et al., 2018). Compared with the isotope-labeling method, the unlabeled quantitative method has a larger dynamic range, wider proteome coverage, and uses simpler experimental protocols. In this study, we investigated the effects of various components extracted from *P. cuspidatum* on laccase production by *T. versicolor* and further determined the mechanisms that drive laccase expression based on omics analyses and bioimaging. Based on the screening of strong laccase inducers, differential protein expression profiles were obtained between the induced group and the control group using LFQ proteomic analysis, which were used to explore the mechanism through which laccase expression is regulated.

In this study, we also observed the effect of emodin on mitochondria through protoplast flow cytometry and confocal laser scanning microscope (CLSM). Filamentous fungi are not suitable for flow cytometry, so the protoplasts of *T. versicolor* can be culture to pass flow cytometry for measuring the effect of emodin on *T. versicolor* mitochondrial membrane potential. Emodin is inherently fluorescent, which facilitated the performance of bioimaging experiments. Fluorescence images of the nanostructure inside the mycelium were obtained, and relative quantitative measurements of the fluorescence signal were performed.

## Results

### Effects of Extracts Using Different Solvents on Laccase Production

**FIGURE 1:**
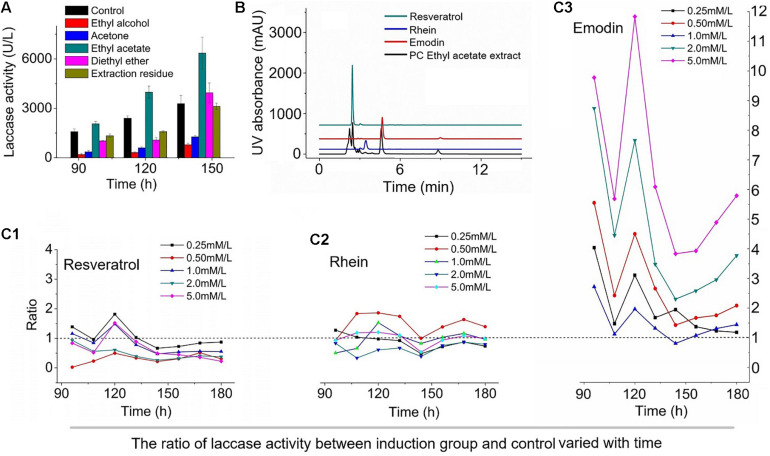
The effect of different components of *Polygonum cuspidatum* on the laccase production of *Trametes versicolor.* **(A)**, The effect of extracts of different polar solvents on the activity of laccase; **(B)**, HPLC analysis of ethyl acetate extract; and the effects of different concentrations of resveratrol **(C1)**, rhein **(C2)**, and emodin **(C3)** on laccase production in *T. versicolor*, The value is the ratio of laccase activity to the control group under the influence of three inducers at different concentrations.

*Polygonum cuspidatum* contains a number of bioactive substances, such as carbohydrates, lignin, resveratrol, and emodin (Chu et al., 2004; Peng et al., 2013). Therefore, the effects of the components in *P. cuspidatum* on laccase production by *T. versicolor* were investigated (Figure 1A). The addition of the ethyl ether extract and extraction residue to the *T. versicolor* medium had no effects on fermentation efficiency, and the highest laccase activity was measured at 6358.1 U⋅L^–1^ following the addition of the ethyl acetate extract, which represented a 93.1% increase compared with the enzymatic activity measured in the control. The addition of the ethanol extract and acetone extract resulted in decreased laccase activity, indicating that the ethyl acetate extract of *P. cuspidatum* contained the prime contributing factor involved in the enhancement of laccase production.

### Evaluation of the Ethyl Acetate Extract of *P. cuspidatum* on Laccase Production

After validating the appropriate extraction solvent, we identified the components found in the ethyl acetate extract of *P. cuspidatum* to determine which factors played major roles in the induction of laccase production. The results of high-performance liquid chromatography (HPLC) showed that resveratrol, rhein, and emodin were the primary components in the *P. cuspidatum* ethyl acetate extract, with concentrations of 49.95, 1.4, and 22.61 μM⋅g^–1^, respectively (Figure 1B). Emodin is known to be a promising multi-biofunctional phytochemical (Li L. et al., 2016), which is abundant in *P. cuspidatum*, as are resveratrol (Lin et al., 2016) and rhein (Feng et al., 2016). To identify the most effective laccase production inducers in the ethyl acetate extract of *P. cuspidatum*, resveratrol, rhein, and emodin are tested individually for their abilities to induce laccase fermentation, at concentrations ranging from 0.25 to 5.0 mM⋅L^–1^ (Feng et al., 2016). As shown in Figure 1C, the addition of emodin to the *T. versicolor* medium produced a notable effect on laccase induction, with enzymatic activity was 4–12 fold that of the control at the additive amount for 5 mM/L. However, rhein had only the slightest effect on laccase production, and resveratrol resulted in the inhibition of laccase production at all tested concentrations compared with the control. The addition of emodin to the supernatant derived from the culture broth obtained from the *T. versicolor* culture resulted in no significant changes in laccase activity (data not shown). This study represents the first report that the phenolic compound emodin can act as an efficient inducer of laccase production in *T. versicolor*.

### 1000-L Pilot Scale-Up Submerged Fermentation

A 1,000-L pilot scale-up submerged fermentation experiment was performed to validate the capabilities of *P. cuspidatum* and emodin to induce laccase production by *T. versicolor*. Using a flask-shaking, batch-fed fermentation technique, the submerged fermentation of *T. versicolor* was performed with the addition of 1% *P. cuspidatum* and 5 mM⋅L^–1^ emodin in a 1,000-L fermenter. Appropriate dissolved oxygen and mass transfer had significant enhancement effects on laccase activity in an industrial bioreactor. The optimization of process controls for the industrial fermented resulted in the control, *P. cuspidatum*, and emodin groups attaining laccase activity values of 200%, 128%, and 289% those measured in 250-ml Erlenmeyer flasks, respectively (Supplementary Figure 1). These results supported the conclusion that certain molecules in TCH could serve as inducers for laccase production.

### Transcriptome Analysis

**FIGURE 2:**
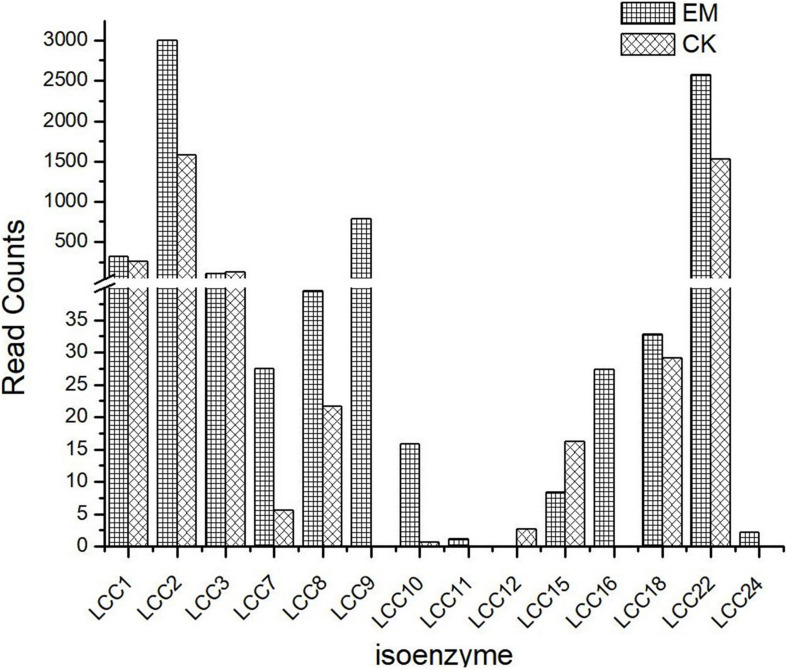
Laccase isoenzyme expression in two groups of strains.

To determine how emodin alters the expression of genes involved in laccase synthesis, eukaryotic non-parametric transcriptomics analysis was performed to establish a database using NCBI BLAST + software (NCBI-blast-2.2.28 + -win32.exe), which resulted in the identification of 58,353 genes which could be detected from the control check (CK) and emodin-induct group (EM) strains (Supplementary Figure 2). These genes were compared against those listed in NCBI’s non-sink protein database, including Swiss-Prot, Protein Information Resource, Protein Research Foundation, and Protein Data Bank, and a total of 14 laccase isozyme genes were identified (Figure 2). As shown in Figure 2, after 6 days of co-cultivation with emodin, the transcription of laccase genes in *T. versicolor* significantly increased. Except for *lcc3*, *lcc12*, and *lcc15*, the mRNA levels of all identified laccase isoenzymes increased in EM comparing with those in CK. Among the upregulated isoenzyme genes, *lcc2* and *lcc22* were associated with the highest expression level and the largest increases in expression level, respectively. The *lcc2* expression level in the EM group was 1.9 times that in the CK group, and the expression level of *lcc22* in the EM group was 1.68 times that in the CK group. In the EM group, the level of *lcc9* expression was high, second only to *lcc2* and *lcc22*, whereas the expression of this isoenzyme could not be detected in the CK group. Because gene expression requires protein translation to be effective, proteomic data analysis is also necessary to explore the effects of emodin on the laccase production in *T. versicolor*.

### Label-Free Differential Proteomics Analysis

To obtain a more precise picture of the effects of gene expression changes, LFQ proteomics was performed based on transcriptome analysis. Respectively, 1,718 and 1,982 proteins were identified in the CK and EM strains, including 1,634 that were common to both groups (Supplementary Figure 3). A total of 223 proteins were identified with large differences in expression between the groups based on standard screening methods, and 29 differentially expressed proteins were identified with fold-changes greater than 2 or less than 0.5 and *P*-values <0.05. These 29 differential proteins were clustered using hierarchical clustering, and the data was displayed in the form of a heat map. Compared with the levels in the CK group, the levels of 15 proteins were increased in the EM group, whereas the levels of 14 proteins decreased. A clear dividing line was observed between the CK and EM groups in the heat map (Supplementary Figure 4).

**FIGURE 3:**
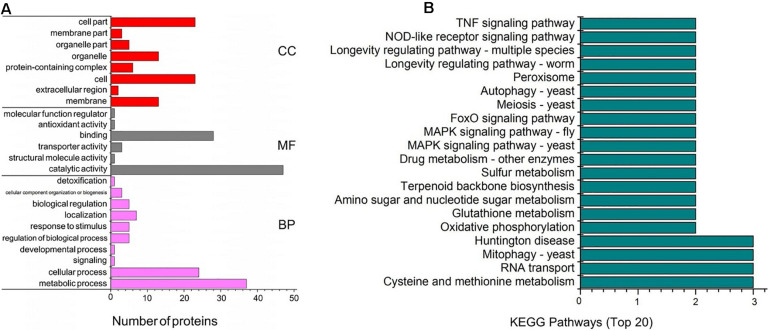
Bioinformatics analysis of differential proteins in *Trametes versicolor*. **(A)** Functional analysis of differential proteins, including the quantity of proteins in secondary function part; **(B)** KEGG pathway enrichment analysis for differentially expressed proteins between the CK and EM bacterial strain.

For insight into the functional categories that were altered between the CK and EM groups, gene ontology (GO) data was used to describes the characteristics of the identified genes and gene products. The comparison and prediction of gene functions using GO analysis are widely used in the field of bioinformatics. GO covers three biological aspects: molecular function (MF), biological process (BP), and cellular component (CC). Within these three aspects, all proteins with significant differences can be divided into 24 secondary annotations. Major BPs associated with the identified differentially expressed proteins included metabolic processes (37 proteins) and cellular processes (24 proteins). In addition, the regulation of biological processes (five proteins), responses to stimuli (five proteins), biological regulation (seven proteins), and localization (five proteins) are well represented among the differentially expressed proteins. The most important MFs included catalytic activity (47 proteins) and binding (28 proteins, Figure 3A). Among the CC categories, the significant differentially expressed proteins were associated with organelles (13 proteins), cells (23 proteins), cellular parts (23 proteins), and membranes (13 proteins, Figure 3A).

To investigate the mechanism through which emodin promotes laccase production in *T. versicolor*, the detected genes were analyzed using the Kyoto Encyclopedia of Genes and Genomes (KEGG) and annotated using KEGG Automatic Annotation Server (KAAS). A total of 120 metabolic pathways were identified, and Figure 3B shows the metabolic pathways associated with the most significant differentially expressed proteins. These metabolic pathways involve redox reactions (mitochondria-yeast, oxidative phosphorylation, glutathione metabolism, and peroxisomes), gene expression regulation [RNA transport, mitogen-activated protein kinase (MAPK) signaling pathways-yeast, MAPK signaling pathways-Drosophila, and forkhead box o (FoxO) signaling pathway], and cell growth division (MAPK signaling pathway-yeast, MAPK signaling pathway-Drosophila, meiosis-yeast, autophagy-yeast, longevity regulatory pathway-worm, and longevity regulatory pathway-multiple species).

### Target Confirmation of Selected Proteins by Parallel Reaction Monitoring (PRM)

**FIGURE 4:**
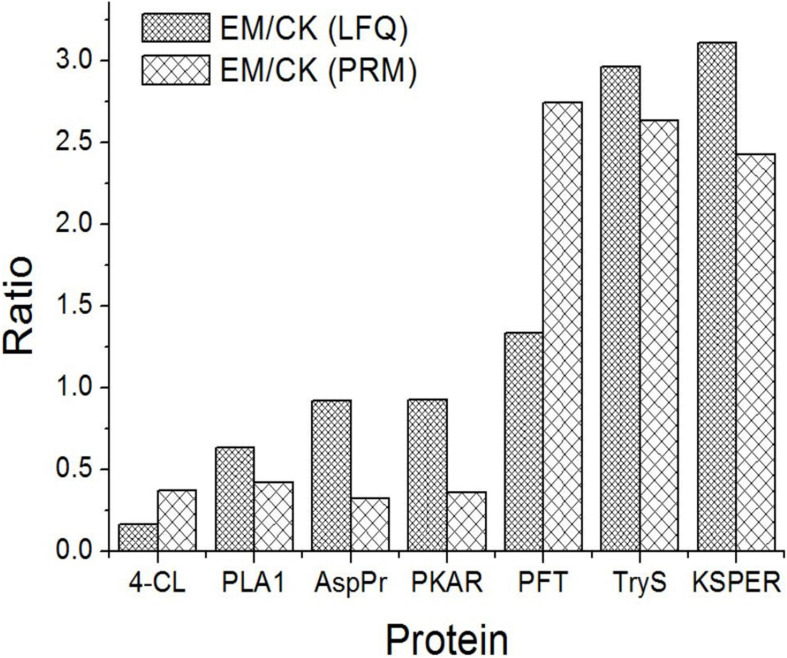
Expression patterns of selected differentially abundance proteins using LFQ analysis and PRM validation.

Motivated by verification of the bioinformatics analysis, PRM was performed to verify the differentially expressed proteins identified by the LFQ. Seven proteins were selected for validation by PRM: 4-coumarate:CoA ligase-like (4-CL), phospholipase A1 (PLA1), aspartyl protease (AspPr), protein kinase A regulatory subunit (PKAR), protein farnesyltransferase subunit beta (PFT), tryptophan synthase (TryS), and PKS-ER domain-containing protein (KSPER). Among these proteins, PFT, TryS, and KSPER had fold-changes above 1.2 (upregulated), and 4-CL, PKAR, AspPr, and PLA2 had fold-changes below 1.0 (downregulated). The PRM analysis indicated that the selected proteins showed similar trends as those identified by the LFQ proteomics analysis, confirming the credibility of the proteomics data (Figure 4).

### Measurement of Mitochondrial Membrane Potential by Protoplast Flow Cytometry

**FIGURE 5:**
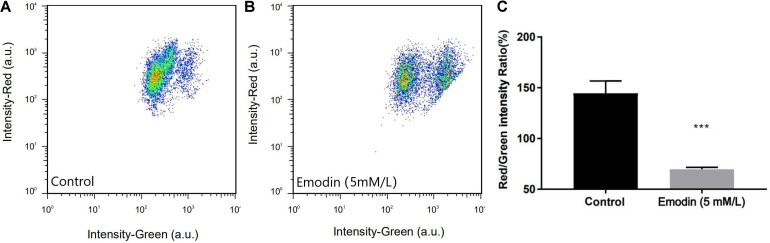
Results of fluorescence distribution for protoplast of control **(A)** and emodin-induction **(B)**. The Red/Green intensity Ratio **(C)** shows the Effect ofemodin on the mitochondrial membrane potential (ΔΨm) in protoplast of *T. versicolor*, it’s significantly lowered compared with the control. (***P < 0.001).

Since the proteomics analysis results main point to mitochondrial metabolism, we measured the changes of the transmembrane potential ΔΨm of protoplast mitochondria so as to verify the effect of emodin on the mitochondria of *T. versicolor*. The JC-1 fluorescent was used to detect whether mitochondrial membrane integrity was damaged. JC-1 will accumulate in the mitochondrial matrix to form a polymer lead to red fluorescence when is high; in other case, they don’t aggregate and fluoresce green. Therefore, the changes in of ΔΨm can be detected by the attribute of fluorescence. In this experiment, after treating with 5 mM/L emodin for 24 h, the protoplasts showed a significant decrease in the ratio of red/green fluorescence, indicating that the mitochondrial membrane potential of the cells decreased in the treatment group, indicating that emodin may be a mitochondrial uncoupling agent (Figure 5).

### Fluorescence Image by CLSM

**FIGURE 6:**
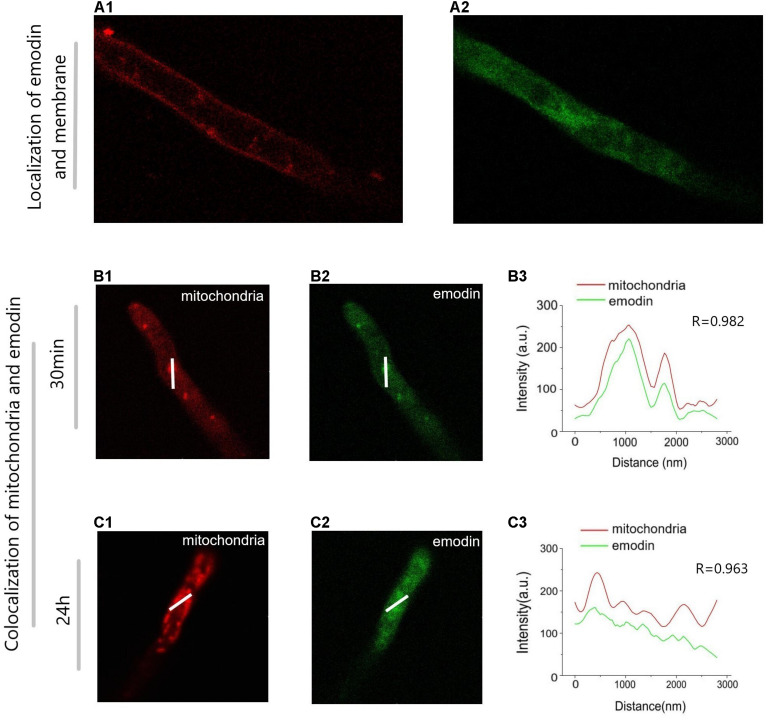
Image of *Trametes versicolor* mycelium infected with emodin shot by confocal laser scanning microscope. The image displays the position of cytomembrane **(A1)** and the fluorescence intensity of emodin Inside the mycelium **(A2)**. It reveals the location of mitochondriais **(B1)** and emodin **(B2)** after 30 min of incubation, the position of mitochondriais **(C1)** and emodin **(C2)** after 24 h and the Intensity profile plot along the selected lines **(B,C)** in images **(B3,C3)**. Emodin has autofluorescence, which the concentration in suspension is 0.5 μmol/mL, cytomembrane and mitochondria are, respectively, stained with Mito Tracker Deep Red and Cell Mask Deep Red for 1 μL/mL.

To study the dynamic changes of emodin in mycelium, a bioimaging experiment was performed (Figure 6). In Figures 6A1,A2, the green and red fluorescence show the locations of emodin and the cell membrane, respectively. We predicted that emodin was able to cross the cell membrane, as the Pearson correlation coefficient between red and green fluorescence was -0.0175. In Figures 6B,C, We selected two groups of fermented liquid: 5 mM/L emodin infected for 0.5 h (Figure 6B) and 24 h (Figure 6C). The green and red fluorescence indicate the localization of emodin and mitochondria, respectively. 30 min after infected by emodin, green and red fluorescence were concentrated in the mycelium, with a high degree of overlap immediately after emodin was added to the mycelium (Figures 6B1–B3). After 24 h, the green fluorescence in the mycelium was scattered, and the fluorescence intensity was slightly reduced (Figures 6C1–C3).

## Discussion

We proposed that emodin stimulates laccase production through the regulation of gene promoter response elements. Laccase gene expression can be promoted by the activation of one or more response elements upstream of the gene promoter. These elements include metal response elements (MREs), which are involved in metal ion response (Faraco, 2003); cAMP-mediated glucose repression sites (creAs) (Piscitelli et al., 2011); nitrogen repression response elements (NIT2s) (Janusz et al., 2013); xenobiotic response elements (XREs) (Rushmore et al., 1990); antioxidant response element (AREs) (Soden and Dobson, 2010); and heat shock response elements (HSE) (Janusz et al., 2013).

Emodin or its metabolites may activate the XRE element upstream of the laccase gene. The gene sequences of many enzymes involved in redox responses, such as cytochrome P450 and glutathione-S transferase (GST), contain upstream XRE elements (Rushmore et al., 1990), which can be activated when the aryl hydrocarbon receptor (AHR) binds to a particular ligand (Abiko et al., 2016). Responsive metabolites of XREs, such as alcohols, acids/ketones, and aromatic compounds, can cause damage to cell growth and development. The XRE associated with the laccase gene promoter region responds to these stimulating substances, initiating the increased expression of the laccase gene to alleviate cellular damage (Xiao et al., 2006). Studies have shown that polycyclic aromatic hydrocarbon compounds, such as quinones and aflatoxin, can activate cytochrome P450 production through an AHR-mediated pathway (Mary et al., 2015; Abiko et al., 2016). As shown in Table 1 shows, cytochrome P450 is overexpressed, which may accelerate the metabolism of emodin (Xing et al., 2018).

**TABLE 1:** Identification of differentially expressed proteins in induced strains (partial).

NR_annotation	Average E	Average C	E/C	*t*-test *p*-value
**Oxidative phosphorylation**				
Cytochrome P450	6884250	1065580	6.460566	0.00038833
Ubiquinol-cytochrome C reductase hinge protein	91502000	208170000	0.439554	0.000922577
Mitochondrial import inner membrane translocase subunit	5483833.333	2689100	2.039282	0.035539778
**Fat metabolism**				
Prenyltransferase and squalene oxidase	12828933.33	4667533.333	2.748547	0.048784295
Lipase C	1316133333	3146333333	0.418307	0.02007442
Protein farnesyltransferase subunit beta	12828933.33	4667533.333	2.748547	0.048784295
**Energy metabolism**				
Glycosyl hydrolases family 18	20294000	43091000	0.470957	0.03729098
Acetyl-CoA synthetase	217385000	14735100	14.752869	0.410862022
Malate synthase	13998600	6067200	2.307259	0.337132896
Phosphoenolpyruvate carboxykinase	584813333.3	259803333.3	2.250985	0.226464407
Beta-glucosidase	1725286667	595020000	2.899544	0.198770664
Glc-6-P isomerase	6319266.667	20192233.33	0.312955	0.11360315
Glyceraldehyde-3-phosphate dehydrogenase	4549600000	12701966667	0.358181	0.082854373
Alpha-galactosidase 3	21777000	44196333.33	0.492733	0.204141001
Cellulase	24450233.33	53375000	0.458084	0.2083071
Glycosyl hydrolases family 15	6075350	13918666.67	0.436489	0.064654334
**Transcriptional regulation and protein modification**				
Zinc-binding dehydrogenase	741790000	305740000	2.426212	0.006570902
Transcription factor	14506666.67	6229966.667	2.32853	0.012676278
CUE domain-containing protein	37493333.33	15602500	2.403034	0.004593804
**Redox reduction**				
Glutathione S-transferase	192853333.3	73178000	2.6354	0.01425529
Mn Superoxide dismutase	175510000	83465000	2.102798	0.016308425
Cu/Zn superoxide dismutase	145762000	71065333.33	2.051099	0.222748309
Aldo-keto reductase 2	22741666.67	9734266.667	2.336249	0.03178134
Catalase	811863496.7	1202640	675.067765	0.373984339
**Metabolism of amino acid and peptide chain**				
Zinc carboxypeptidase	12315200	4655600	2.645244	0.034308689
Tryptophan synthase	1381903333	3743233333	0.369174	0.004840908
**Others**				
Heat shock protein SSB1	176745850	–	–	–
Primary-amine oxidase	27834250	–	–	–
Histone-fold-containing protein	150165000	–	–	–
Alcohol dehydrogenase	248897500	–	–	–
O-acetylhomoserine (thiol)-lyase	218074950	–	–	–
4-hydroxyacetophenone monooxygenase	–	44245000	–	–
Isochorismatase hydrolase	–	3543000	–	–
Alpha/beta-hydrolase	–	2625050	–	–
Sucrose non-fermenting 1	–	2590500	–	–
MFS general substrate transporter	–	3977650	–	–

Based on the nature and functions of the differentially expressed proteins identified between the CK and EM groups, we proposed that the addition of emodin induced *T. versicolor* laccase secretion by affecting energy metabolism. Compared with the CK group, the expression of several catabolic enzymes was upregulated in the EM group. Succinyl-CoA synthetase beta subunit and malate synthase are tricarboxylic acid (TCA) cycle-related proteins, and their upregulation is associated with the acceleration of ATP synthesis. The expression levels of alcohol dehydrogenase, aldehyde dehydrogenase, and acetyl-CoA synthetase were all upregulated, The first two can oxidize ethanol to acetic acid and Acetyl-CoA synthetase catalyzes acetic acid with coenzyme A to synthesize acetyl-CoA during energy metabolism. Above indicating that anaerobic respiration may appear in the mycelium and accelerate the consumption of carbon sources. Table 1 shows that zinc carboxypeptidase, amidohydrolase, and ornithine aminotransferase were also altered, suggesting that emodin can enhance protein and amino acid metabolism. The upregulation of these three enzymes indicated that the glucose content of the cell species was too low, requiring the mycelium to consume proteins and amino acids to supplement energy requirements. This evidence suggested that emodin, as a mitochondrial uncoupler, reduces the level of ATP in cells (Sugiyama et al., 2019) and promotes glucose oxidation (Jiang et al., 2019). As glucose levels decrease, the CreA protein is released from the CreA element, activating the expression of downstream genes, including laccase. Laccase is a secondary metabolic protein, the expression of which is activated when glucose contents are low (Hahn Schneider et al., 2018). In fermentation experiments, after the easy-to-use carbon sources, such as glucose, are consumed (72–96 h), the laccase expression level increases (Figure 1C3).

The downregulation of some anabolic enzymes (such as tryptophan synthase, cytokinin-activated protein kinase, 14-3-3 protein, and ATP-dependent DNA helicase) was also observed, which inhibited mycelium growth and division (Figure 3). As a low-energy signal, low ATP levels spontaneously trigger the assembly of the adenosine 5’-phosphate adenosine-activated protein kinase (AMPK) complex (Zhang et al., 2013), which promotes catabolism and inhibits anabolism (Huang et al., 2015). Therefore, the addition of emodin may activate the AMPK pathway, which, in turn, activates ATP production and inhibits ATP consumption. The verification experiment also demonstrated that the biomass of *T. versicolor* mycelium decreased in the presence of emodin compared with that of the control group. In the EM group, the 4-CL content was reduced. 4-CL is involved in phenylpropane anabolic metabolism and is an essential enzyme during the synthesis of secondary metabolites, such as flavonoids (Praveen et al., 2019). As a key enzyme involved in an anabolic pathway that consumes ATP, the expression of 4-CL may be inhibited by AMPK.

Emodin can stimulate the oxidative stress response in the mycelium. Studies have shown that emodin increases the concentration of reactive oxygen species (ROS) *in vivo* (Li X. et al., 2016). Compared with the CK group, the expression of some redox enzymes in the EM group increased, including superoxide dismutase (SOD), peroxidase (POD), and catalase (CAT). The expression of these enzymes is induced when the intracellular ROS concentration increases, and these enzymes reduce ROS concentrations to alleviate cellular damage induced by ROS and electrophilic substances. The ARE is activated in response to increased intracellular ROS (Soden and Dobson, 2010), binding to the nuclear transcription factor nuclear erythroid 2-related factor 2 (Nrf2), which is a key transcription factor involved in the cellular antioxidant stress system. Emodin has previously been shown to activate the Nrf2/ARE signaling pathway (Song et al., 2019), which may be mediated by the AMPK pathway. AMPK catalyzes the phosphorylation of serine 550 in the Nrf2 protein, promoting the nuclear translocation of Nrf2 (Joo et al., 2016), which then induces the expression of oxidative stress-related enzymes, such as SOD, CAT, and other oxidases, including laccase.

Emodin may stimulate proton leakage to reduce ATP levels in cells (Sugiyama et al., 2019). As a lipophilic and weakly acidic polycyclic aromatic hydrocarbons, emodin can pass through the cell membrane and the outer mitochondrial membrane. Emodin is more likely to bind protons in a proton-rich environment (such as outside of the inner mitochondrial membrane), whereas emodin promotes the release of H^+^ in a neutral environment (Sugiyama et al., 2019). The rate of ATP synthesis synchronizes with the electrochemical H^+^ gradient on both sides of the mitochondrial inner membrane, activating the synthesis of energy metabolism-related enzymes to increase ATP production.

As shown in Figure 5, **30** min after emodin was added to the mycelium, emodin-associated green fluorescence was observed to be concentrated in the mitochondrial region, which may be related to the protophilia of its carbonyl group. As emodin gradually degraded over the following 24 h, the fluorescence that was initially concentrated in the mitochondrial region dissipated, and the fluorescence intensity decreased. Based on these confocal laser scanning experiments and the omics analysis, emodin was hypothesized to have been attracted to the H^+^-rich environment outside of the inner mitochondrial membrane, where it reduced the mitochondrial membrane potential until it was degraded by secondary metabolic enzymes, including included laccase, SOD, CYP450, and GSTs.

Emodin or its degradative products is thought to bind AHRs, which stimulate XREs to induce the expression of Nrf2 and some redox-related enzymes, which degrade these toxic substances including emodin. Simultaneously, emodin reduces the efficiency of mitochondrial operations by disrupting the mitochondrial membrane potential, promoting glucose consumption. Laccase production is induced to meet the demand for carbon resources. Low ATP levels also activate the AMPK pathway, which can phosphorylate Nrf2 and bind with AREs in the nucleus to synthesizes a series of oxidoreductases, including laccase, to reduce ROS generated in response to emodin (Figure 6). These findings allow us to infer the influence of emodin in *T. versicolor* at the molecular level, providing a simple assessment method for evaluating other high-yield laccase induction conditions and the regulation of secondary metabolism by other factors, such as polysaccharides and triterpenes.

## Conclusion

**FIGURE 7:**
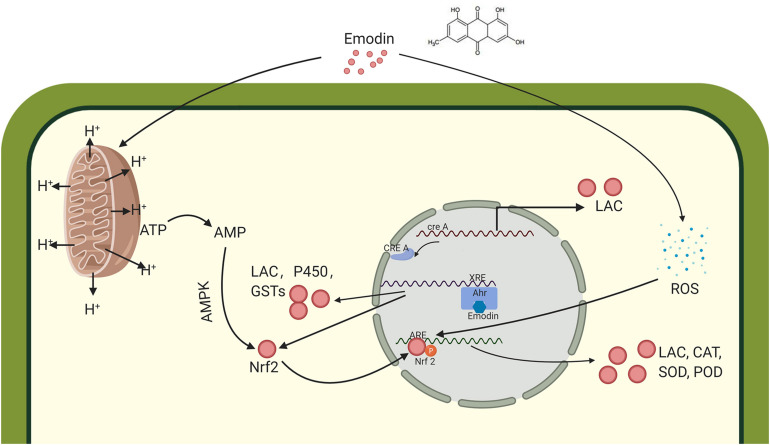
Schematic illustration of the induction of laccase by emodin in *Trametes versicolor*.

In this study, *P. cuspidatum* called for obvious acceleration of laccase production from *T. versicolor*. Emodin was confirmed as a key inducer within active ingredients. Scale-up fermentation results demonstrated that using medicinal herb residues as natural inducer could reduce the cost for the process of submerged fermentation and enhance the laccase production. Using LFQ proteomics analysis to analyze the molecular mechanisms, it showed that these identified differential proteins function in a variety of ways, including metabolic processes, regulatory biological processes, catalytic activity, and binding. At the same time, these differential proteins are involved in a variety of metabolic pathways, such as Oxidative phosphorylation, Glutathione metabolism and Peroxisome, MAPK signaling pathway. Finally, it determined that these proteins are involved in the efficient expression of laccase (Figure 7). This study compares different proteins, determined the effect of emodin on the expression and its molecular mechanism of laccase in *T. versicolor*. The result provides a feasible strategy for strain selection and industrial production process optimization, and then expands the application of laccase in environmental protection, pollutant degradation, food industry and biocatalysis.

## Data Availability Statement

The data presented in the study are deposited in the (http://proteomecentral.proteomexchange.org/cgi/GetDataset) repository, accession number (1-20210421-40601).

## Author Contributions

LW and XL designed the experiments and completes experiment and data collation of the Fermentological research, YP and ZZ contributed the omics experiment and data analysis. XT and LZ provided the data of fluorescence image and flow cytometry experiment. RY furnish the final modification of the overall experimental plan of the article.

## Conflict of Interest

LW and XL were employed by the company Hefei Tingxiandu Biological Technology Co., Ltd. The remaining authors declare that the research was conducted in the absence of any commercial or financial relationships that could be construed as a potential conflict of interest.

## References

[B1] Abiko Y. Lin F. Y. Lee H. Puga A. Kumagai Y. (2016). Quinone-mediated induction of cytochrome P450 1A1 in HepG2 cells through increased interaction of aryl hydrocarbon receptor with aryl hydrocarbon receptor nuclear translocator. *J. Toxicol. Sci.* 41 775–781. 10.2131/jts.41.775 27853106

[B2] Bains A. Chawla P. (2020). *In vitro* bioactivity, antimicrobial and anti-inflammatory efficacy of modified solvent evaporation assisted *Trametes versicolor* extract. *3Biotech* 10:404. 10.1007/s13205-020-02397-w 32903990 PMC7447717

[B3] Bettin F. Montanari Q. Calloni R. Gaio T. A. Silveira M. M. Dillon A. J. P. (2009). Production of laccases in submerged process by *Pleurotus sajor-caju* PS-2001 in relation to carbon and organic nitrogen sources, antifoams and Tween 80. *J. Ind. Microbiol. Biotechnol.* 36 1–9. 10.1007/s10295-008-0463-1 18758836

[B4] Chu Q. Peng Y. Ye J. (2004). Determination of Active Ingredients of *Polygonum cuspidatum* Sied. et Zucc. by capillary electrophoresis with electrochemical detection. *Electroanalysis* 16 1434–1438. 10.1002/elan.200302968

[B5] Faraco V. (2003). Metal-responsive elements in *Pleurotus ostreatus* laccase gene promoters. *Microbiology* 149 2155–2162. 10.1099/mic.0.26360-0 12904555

[B6] Feng J. F. Ren H. Z. Gou Q. F. Zhu L. (2016). Comparative analysis of the major constituents in three related polygonaceous medicinal plants using pressurized liquid extraction and HPLC-ESI/MS. *Anal. Methods* 8 1557–1564. 10.1039/C5AY02941D

[B7] Gonzalez J. C. Medina S. C. Rodriguez A. Osma J. F. Sánchez O. F. (2013). Production of *Trametes pubescens* laccase under submerged and semi-solid culture conditions on agro-industrial wastes. *PLoS One* 8:e73721. 10.1371/journal.pone.0073721 24019936 PMC3760920

[B8] Hahn Schneider W. D. Fontana R. C. Mendonca S. De Siqueira F. G. Pinheiro Dillon A. J. Camassola M. (2018). High level production of laccases and peroxidases from the newly isolated white-rot basidiomycete *Marasmiellus palmivorus* in a stirred-tank bioreactor in response to different carbon and nitrogen sources. *Process Biochem.* 69 1–11. 10.1016/j.procbio.2018.03.005 33582009

[B9] Huang B. P. Lin C. H. Chen H. M. Lin J. T. Cheng Y. F. Kao S. H. (2015). AMPK activation inhibits expression of proinflammatory mediators through downregulation of PI3K/p38 MAPK and NF-κB signaling in murine macrophages. *DNA Cell Biol.* 34 133–141. 10.1089/dna.2014.2630 25536376

[B10] Iimura Y. Sonoki T. Habe H. (2018). Heterologous expression of *Trametes versicolor* laccase in *Saccharomyces cerevisiae*. *Protein Expr. Purif.* 141 39–43. 10.1016/j.pep.2017.09.004 28918197

[B11] Janusz G. Kucharzyk K. H. Pawlik A. Staszczak M. Paszczynski A. J. (2013). Fungal laccase, manganese peroxidase and lignin peroxidase: gene expression and regulation. *Enzyme Microb. Technol.* 52 1–12. 10.1016/j.enzmictec.2012.10.003 23199732

[B12] Jiang H. Jin J. Duan Y. Xie Z. Li Y. Gao A. (2019). Mitochondrial uncoupling coordinated with PDH activation safely ameliorates hyperglycemia via promoting glucose oxidation. *Diabetes Metab. Res. Rev.* 68 2197–2209. 10.2337/db19-0589 31471292

[B13] Joo M. S. Kim W. D. Lee K. Y. Kim J. H. Koo J. H. Kim S. G. (2016). AMPK facilitates nuclear accumulation of Nrf2 by phosphorylating at serine 550. *Mol. Cell. Biol.* 36 1931–1942. 10.1128/MCB.00118-16 27161318 PMC4936058

[B14] Kuhar F. Papinutti L. (2014). Optimization of laccase production by two strains of *Ganoderma lucidum* using phenolic and metallic inducers. *Rev. Argent. Microbiol.* 46 144–149. 10.1016/S0325-7541(14)70063-X 25011599

[B15] Li L. Song X. Yin Z. Jia R. Li Z. Zhou X. (2016). The antibacterial activity and action mechanism of emodin from *Polygonum cuspidatum* against *Haemophilus parasuis in vitro*. *Microbiol. Res.* 139–145. 10.1016/j.micres.2016.03.008 27242151

[B16] Li X. Wang H. Wang J. Chen Y. Yin X. Shi G. (2016). Emodin enhances cisplatin-induced cytotoxicity in human bladder cancer cells through ROS elevation and MRP1 downregulation. *BMC Cancer* 16:578. 10.1186/s12885-016-2640-3 27485374 PMC4971704

[B17] Li Y. Jiang G. Niu J. Ying W. Hu L. (2009). Laccase-catalyzed oxidation of organic pollutants in water. *Prog. Chem.* 21 2028–2036. 10.1016/S1874-8651(10)60079-8

[B18] Lin J. A. Kuo C. H. Chen B. Y. Li Y. Liu Y. C. Chen J. H. (2016). A novel enzyme-assisted ultrasonic approach for highly efficient extraction of resveratrol from *Polygonum cuspidatum*. *Ultrason. Sonochem.* 32 258–264. 10.1016/j.ultsonch.2016.03.018 27150769

[B19] Mary V. S. Valdehita A. Navas J. M. Rubinstein H. R. Fernández-Cruz M. L. (2015). Effects of aflatoxin B1, fumonisin B1 and their mixture on the aryl hydrocarbon receptor and cytochrome P450 1A induction. *Food Chem. Toxicol.* 75 104–111. 10.1016/j.fct.2014.10.030 25449202

[B20] Mate D. M. Alcalde M. (2016). Laccase: a multi-purpose biocatalyst at the forefront of biotechnology. *Microb. Biotechnol.* 10 1457–1467. 10.1111/1751-7915.12422 27696775 PMC5658592

[B21] Patel N. Shahane S. Shivam, Majumdar R. Mishra U. (2018). Mode of action, properties, production, and application of laccase: a review. *Recent Pat. Biotechnol.* 13 19–32. 10.2174/1872208312666180821161015 30147019

[B22] Peng W. Qin R. Li X. Zhou H. (2013). Botany, phytochemistry, pharmacology, and potential application of *Polygonum cuspidatum* Sieb.et Zucc.: a review. *J. Ethnopharmacol.* 148 729–745. 10.1016/j.jep.2013.05.007 23707210

[B23] Pinheiro V. E. Michelin M. Vici A. C. Almeida P. Z. D. Polizeli M. D. L. T. D. M. (2020). *Trametes versicolor* laccase production using agricultural wastes: a comparative study in Erlenmeyer flasks, bioreactor and tray. *Bioprocess Biosyst. Eng.* 43 507–514. 10.1007/s00449-019-02245-z 31709470

[B24] Piscitelli A. Giardina P. Lettera V. Pezzella C. Faraco V. (2011). Induction and transcriptional regulation of laccases in fungi. *Curr. Genomics* 12 104–112. 10.2174/138920211795564331 21966248 PMC3129044

[B25] Praveen A. Vidushi M. Lakshmi J. V. Rekha C. Nitika K. Bedi Y. S. (2019). Characterization of the gene encoding 4-coumarate:CoA ligase in *Coleus forskohlii*. *J. Plant Biochem. Biotechnol.* 28 203–210. 10.1007/s13562-018-0468-4

[B26] Rangabhashiyam S. Anu N. Selvaraju N. (2013). The significance of fungal laccase in textile dye degradation -a review. *Res. J. Chem. Environ.* 17 88–95. 10.1007/s11136-012-0235-2 22833152

[B27] Rushmore T. H. King R. G. Paulson K. E. Pickett C. B. (1990). Regulation of glutathione S-transferase Ya subunit gene expression: identification of a unique xenobiotic-responsive element controlling inducible expression by planar aromatic compounds. *Proc. Natl. Acad. Sci. U.S.A.* 87 3826–3830. 10.1073/pnas.87.10.3826 2160079 PMC53996

[B28] Shukla V. Asthana S. Yadav S. Rajput V. S. Tripathi A. (2020). Emodin inhibited NADPH-quinone reductase competitively and induced cytotoxicity in rat primary hepatocytes. *Toxicon* 188 117–121. 10.1016/j.toxicon.2020.10.018 33122156

[B29] Soden D. M. Dobson A. D. W. (2010). The use of amplified flanking region-PCR in the isolation of laccase promoter sequences from the edible fungus *Pleurotus sajor-caju*. *J. Appl. Microbiol.* 95 553–562. 10.1046/j.1365-2672.2003.02012.x 12911704

[B30] Song C. Liu B. Xu P. Ge X. Zhang H. (2019). Emodin ameliorates metabolic and antioxidant capacity inhibited by dietary oxidized fish oil through PPARs and Nrf2-Keap1 signaling in Wuchang bream (*Megalobrama amblycephala*). *Fish Shellfish Immunol.* 94 842–851. 10.1016/j.fsi.2019.10.001 31585245

[B31] Sugiyama Y. Shudo T. Hosokawa S. Watanabe A. Kakizuka A. (2019). Emodin, as a mitochondrial uncoupler, induces strong decreases in adenosine triphosphate (ATP) levels and proliferation of B16F10 cells, owing to their poor glycolytic reserve. *Genes Cells* 24 569–584. 10.1111/gtc.12712 31234244

[B32] Widsten P. Kandelbauer A. (2008). Laccase applications in the forest products industry: a review. *Enzyme Microb. Technol.* 42 293–307. 10.1016/j.enzmictec.2007.12.003

[B33] Xiao Y. Z. Hong Y. Z. Li J. F. Hang J. Tong P. G. Fang W. (2006). Cloning of novel laccase isozyme genes from *Trametes* sp. AH28-2 and analyses of their differential expression. *Appl. Microbiol. Biotechnol*. 71 493–501. 10.1007/s00253-005-0188-2 16283298

[B34] Xing Y. Wang L. Youcai C. Yi Z. Hu L. (2018). Pharmacokinetic studies unveiled the drug-drug interaction between trans-2,3,5,4’-tetrahydroxystilbene-2-O-β-d-glucopyranoside and emodin that may contribute to the idiosyncratic hepatotoxicity of Polygoni Multiflori Radix. *J. Pharm. Biomed. Anal.* 164 672–680. 10.1016/j.jpba.2018.11.034 30472586

[B35] Yang H. Sun H. Zhang S. Wu B. Pan B. (2015). Potential of acetylacetone as a mediator for *Trametes versicolor* laccase in enzymatic transformation of organic pollutants. *Environ. Sci. Pollut. Res.* 22 10882–10889. 10.1007/s11356-015-4312-2 25772881

[B36] Zdarta J. Antecka K. Frankowski R. Zgoa-Grzekowiak A. Jesionowski T. (2017). The effect of operational parameters on the biodegradation of bisphenols by *Trametes versicolor* laccase immobilized on *Hippospongia communis* spongin scaffolds. *Sci. Total Environ.* 615 784–795. 10.1016/j.scitotenv.2017.09.213 28992503

[B37] Zhang Y.-L. Guo H. Zhang C.-S. Lin S.-Y. Yin Z. Peng Y. (2013). AMP as a low-energy charge signal autonomously initiates assembly of AXIN-AMPK-LKB1 complex for AMPK activation. *Cell Metab.* 18 546–555. 24093678 10.1016/j.cmet.2013.09.005

[B38] Zixuan Z. Nannan L. Li L. Binghui H. Yasuo I. Feng L. (2018). Label-free differentially proteomic analysis of interspecific interaction between white-rot fungi highlights oxidative stress response and high metabolic activity. *Fungal Biol.* 122 774–784. 10.1016/j.funbio.2018.04.005 30007428

