# Supplementary material for: Mechanism of Laccase Induction via Emodin in Trametes versicolor
Source: Front Bioeng Biotechnol. 2021 May 18;9:653800. doi: 10.3389/fbioe.2021.653800 (PMC8171328; doi:10.3389/fbioe.2021.653800)
Supplement: Supplementary file 1 [file Data_Sheet_1.docx]

**Laccase induction via emodin and its regular mechanism in *Trametes versicolor* evaluated by super-resolution nanoscopy**

*Lin Wang*^a,b†^, Xuecai Luo^b†^, Yu Pan^a^, Zai Zheng^a^, Ruochun Yin*^a^, Xiaohe Tian^a^, Liang Zhang^c^*

*^a^ School of Life Sciences, Anhui University, Hefei, Anhui 230601, China.*

*^b^ Hefei Tingxiandu BioTec Co., Ltd, Hefei, Anhui 230088, China.*

*^c^National Engineering Laboratory for Cereal Fermentation Technology, Jiangnan University, Wuxi, 214122, China.*

*^*^Correspondence to: wanglin@ ahu.edu.cn， rcyin@ahu.edu.cn*

† These authors contributed equally.

*Correspondence to: [wanglin@ustc.edu.cn](#mailto:sujihu@ustc.edu.cn), [rcyin@ahu.edu.cn](#mailto:rcyin@ahu.edu.cn)

**Table of Contents**

[Chemicals and microorganisms 3](#_Toc11539)

[Culture medium and growth conditions 3](#_Toc3917)

[Effect of crude extracts from *P. cuspidatum* on laccase production 3](#_Toc23070)

[Laccase assay 3](#_Toc7459)

[HPLC identification and quantification 4](#_Toc16858)

[Industrial-scale fermentation condition 4](#_Toc16846)

[Bioinformatics Analysis of Eukaryotic non-ginseng transcriptome 4](#_Toc4434)

[LFQ proteomic analysis 5](#_Toc10135)

[Parallel reaction monitoring (PRM) for target confirmation 5](#_Toc15754)

[Positioning of emodin by STED 6](#_Toc31712)

[Statistical analysis 6](#_Toc24830)

[Figure S1. 7](#_Toc12088)

[Figure S2. 7](#_Toc29182)

[Figure S3. 8](#_Toc28350)

[Figure S4. 8](#_Toc246)

[Figure S5. 9](#_Toc27090)

# Chemicals and microorganisms

Resveratrol, rhein, emodin and indole were obtained from Sigma-Aldrich (USA). All other chemicals were analytical grade and were purchased from Tianjin Yongda Chemical Reagents Company Limited (Tianjin, China).The *P. cuspidatum* was purchased from Chinese medicines shop (Tong Rentang pharmacy LMT). *T. versicolor* (CICC 14020) were purchased from China Center of Industrial Culture Collection. The stock cultures of *T. versicolor* were maintained on potato dextrose agar (PDA) slants at 4℃ after subculturing at 30℃ for evey 2-3 weeks.

# Culture medium and growth conditions

The *T. versicolor* was first activated in PDB medium (potato extract, 200 g∙L^-1^ , glucose, 20 g∙L^-1^, pH 5.5) in a shaker at 150 rpm, 30℃ for 3 days. Then the pelleted mycelium (5 ml, ~2 mm in diameter) were transferred into 70 mL liquid medium, which prepared for laccase production containing the following (per litre): glucose, 10g; wheat bran, 10g; soybean flour, 10g; KH_2_PO_4_, 7.35mM. This medium was just used as the control. When it comes to evaluate the effect of the *P. cuspidatum* for laccase production, it was added with a dosage of 10 g∙L^-1^ as well as all the main components remained consistent with the control medium. Initial pH of the liquid medium was adjusted to 5.5 with 4N H_2_SO_4_ or 4M NaOH and autoclaved at 121℃ for 20 min. Flasks were incubated at 30℃ and 150 rpm for 6-9 days.

# Effect of crude extracts from *P. cuspidatum* on laccase production

The *P. cuspidatum* power (20g) was extracted with 60 ml of the following organic solvents: ethyl acetate, ethanol, acetone and ethyl ether, respectively at 30℃ for three times of each. The residue was subjected to further extraction in 60ml of boiling water and used as a positive control. The extract solvents and positive control were then evaporated by vacuum rotary evaporator at 40℃, and all the dry residue were resuspended in 10 mL water and used as the crude organic extracts. 5 mL of the extracts (equal to the *P. cuspidatum* dosage of 10 g∙L^-1^) was added to 1 L of the control medium and used for the laccase fermentation.

# Laccase assay

Laccase activity was quantified spectrophotometrically with 0.6 ml of the propriety concentration culture supernatants pre-centrifuged (12000rpm, 5min) were added into a graduated test tube, 2.4 ml pH 4.5 succinic acid–sodium hydroxide buffer solution (containing 1 mmol/L guaiacol) at 30 ℃ for 30 min([Jiang et al., 2011](#_ENREF_13)). One unit of laccase activity was defined as the amount of enzyme required to oxidize 1 mmol of guaiacol min^-1^ ∙L^-1^.

# HPLC identification and quantification

The ethyl acetate extract of *P. cuspidatum* were analyzed using an Agilent HPLC 1100 instrument equipped with a Waters SunFire C18 column (5 um × 4.6 mm × 150 mm) and ultraviolet detector (UV detector), operating at 30℃. The injection volume was 10 ul and optimum separation was achieved with a binary mobile phase at a flow-rate of 1.0 mL∙min^-1^. The mobile phase was composed by solvent A: acetonitrile and solvent B: water (A : B = 60 : 40) for 15min and the samples were analyzed in triplicate. The extract was identified by comparisons of the retention time and UV-visible spectral data with those of authentic compounds and quantification was performed from calibration curves that were obtained using model compounds.

# Industrial-scale fermentation condition

Industrial-scale fermentation was performed in 1000 L automatic mechanical mixing and ventilating fermenter (BIOTECH-400JS, Baoxing, Shanghai). The liquid loading coefficient is 70%, and the amount of inoculation was 7.5%. The operating parameter are maintained at 30 ℃, 120 rpm，0.5 vvm aeration rate and 0.08 MPa in the whole process of fermentation.

# Bioinformatics Analysis of Eukaryotic non-ginseng transcriptome

*T. versicolor* cells induced by emodin and control without inducer were divided into two samples which includes 3 parallel samples respectively for transcriptome and proteome analysis. In [omics analysis](https://kns.cnki.net/KNS8/Detail/RedirectScholar?flag=TitleLink&tablename=SJPDLAST&filename=SJPD298AA61747796727B27726F7C3FF7A84), seed culture medium (glucose 20g·L^-1^, wheat bran 20g·L^-1^, soybean flour 20g·L^-1^, KH_2_PO_4_, 22.06mM·L^-1^) was cultived in shaking flasks at 150 rpm at 30 ℃ for 5 days. The pellets (about 2mm in diameter) were obtained and transferred to 150 ml liquid fermentation medium (200g·L^-1^ potato extract) as the control check (CK). 8mg emodin was added to the CK for 150 rpm, 6 days, as emodin-induct group (EM).

Operating procedures of transcriptome analysis: 1) Library construction. mRNA was randomly interrupted by adding mRNA, enriched with magnetic beads with Oligo (dT) to fragmentation buffer. Using mRNA as template, the first cDNA chain was synthesized with six base random primers (random hexamers), and then buffer, dNTPs, RNase H and DNA p were added. The second cDNA chain was synthesized by olymerase I, and the double-stranded cDNA purified by AMPure XP beads was repaired at the end, then the A tail was added in sequenced, for next the of size fragment was selected with AMPure XP beads. Finally, the cDNA library was obtained by PCR enrichment. 2) The quality control of the library was carried out by using Qubit2.0 to dilute the library to 1 ng/l, and then take Agilent 2100 to pair the library. Insert size detection, the detection results meet the requirements before the computer sequencing. 3) On the computer test, different libraries were separated base on the target data volume, sequencing with Illumina HiSeq platform.

# LFQ proteomic analysis

1) TCA/Acetone Precipitation and SDT Lysis. The samples were frozen in liquid nitrogen and ground with pestle and mortar. Five times the volume of TCA/acetone (1:9) was added to the powder and mixed by vortex. Keep the mixture at-20 ℃ for 4 h and centrifuge at 4℃ with 6000 g for 40 minutes. Discard the liquid; add pre-cooled acetone and wash it three times; air dry the sediment; add 30 times the volume of SDT buffer to 20-30mg powder, mix and boil for 5 minutes; ultrasonic treatment of the lysate, then boil for 15 minutes; centrifuge at 14000g for 40 minutes After that, the culture fluid was filtered with 0.22 μ m filter. Take BCA protein determination kit (Bio-Rad,USA) to quantitative filtrate. Then stored the samples at-80 ℃. 2) SDS-PAGE. The 20 μ g protein of each sample was mixed with 5 times of buffer to boiled for 5 minutes, then proteins were separated on 12.5% SDS-PAGE gel (constant current 14 Ma, 90 minutes). The protein bands were observed by Coomassian blue R-250 staining. 3) Filter-aided sample preparation (FASP Digestion). 200 μ/g protein of each sample was added into 30 μ/l SDT buffer. (4% SDS, 100mm DTT, 150mM Tris-HCl pH 8. 0). Using repeated ultrafiltration (Microcon unit, 10kD), UA buffer (8m urea, 150mM Tris-HCl pH8.0) to remove decontamination agent, DTT and other low molecular weight components, then 100 μl iodoacetamide (100mM IAA in UA buffer) was added to block the reduced cysteine residues and the samples were incubated in the dark for 30 minutes. Wash the filter membrane three times with 100 μ lUA buffer, use 100 μ/l 25 mm NH_4_ HCO_3_ buffer washed twice hereafter. Finally, the protein suspension was digested overnight with 4 μg trypsin (Promega) in 40 μl 25mm NH_4_HCO_3_ buffer at 37 ℃, and the peptides were collected as filtrate. The peptides of each sample were desalted on C18 column (standard density), concentrated by vacuum centrifugation and reconstituted in 40 µl of 0.1% (v/v) formic acid. Through the UV spectrum at 280nm The density estimated peptide content was estimated. 4) LC-MS / MS analysis. LC-MS / MS analysis were carried out on the Q Exactive mass spectrometer (Thermo Scientific). The mass spectrometer was operated in positive ion mode. MS data was acquired using a data-dependent top10 method dynamically choosing the most abundant precursor ions from the survey scan (300–1800 m/z) for high energy collision dissociation (HCD) fragmentation. Dynamic exclusion duration was 40.0 s. Survey scans were acquired at a resolution of 70,000 at m/z 200 and resolution for HCD spectra was set to 17,500 at m/z 200, and isolation width was 2 m/z.. Normalized collision energy was 30 eV and the underfill ratio, which specifies the minimum percentage of the target value likely to be reached at maximum fill time, was defined as 0.1%. The instrument was run with peptide recognition mode enabled.

# Parallel reaction monitoring (PRM) for target confirmation

To verify the protein expression levels obtained by LFQ , the expression levels of selected differentially abundant proteins (unique peptides≥2, fold change≥1.2) were further quantified by PRM analysis. Peptides were prepared base on proteomics analysis, and a normal peptide was spiked in each sample as internal standard reference. The enzymatic peptides were desalted, lyophilized and reconstituted. The chromatographic separation was performed by the nanoliter velocity HPLC system Easy nLC, for using one-hour liquid chromatography gradients with acetonitrile ranging from 5 to 30% over 45 min. PRM analysis was performed by Q-Exactive HF mass spectrometer (Thermo Scientific), while the mass spectrometer was operated in the positive ion mode with the following parameters: The full MS1 scan was acquired at a resolution of 60,000 (at 200 m/z), an automatic gain control (AGC) target value of 3.0 × 10^−6^, and a maximum ion injection time of 200 ms. Intact MS scans were followed by 20 PRM scans at 30,000 resolution (at 200 m/z) with AGC at 3.0×10^−6^ and a maximum injection time of 120 ms. The targeted peptides were isolated within a 1.6 Th window and the ion activation and dissociation were performed at normalized a collision energy of 27 in higher energy dissociation collision cells. The raw data were analyzed using Skyline software(MacCoss Lab, University of Washington) .

# Measurement of mitochondrial membrane potential by protoplast flow cytometry

Culture medium of *T. versicolor* spores, contained (per litre): glucose 20g; (NH_4_)_2_SO_4_ 5g; KH_2_PO_4_, 15g; MgSO_4_ 0.6g;CaCl_2_, FeSO_4_, 0.005g; MnSO_4_, 0.0016g; ZnSO_4_, 0.0014g; CoCl_2_, 0.002g; pH5.5 for about 20h to induce it germination, then, enzymatic hydrolysis solution (contain: sorbitol 1.2 mol/L; K_3_PO_4_ 10mmol/L; Lysing Enzymes 10g/L; pH 5.8 ) subject to prepare protoplasts. 24 hours after drugging protoplasts with emodin, a Mitochondrial Membrane Potential Assay Kit was used to measure the mitochondrial membrane potential according to the manufacturers instruction. The control and emodin-invade protoplasts were incubated with JC-1 for 15 min at 37℃ in dark. The stained cells were washed with PBS twice and 500 μL of working solution was added to each well. Then the cells were immediately analyzed by flow cytometry (BD Biosciences company, USA) for at least 10,000 events. JC-1 aggregates in the polarized mitochondrial matrix and forms J-aggregates, which emit red fluorescence at 595 nm when excited at 525 nm.

# Positioning of emodin by CLSM

The *T. versicolor* were cultivated in 70 mL liquid medium which contain the following (per litre): glucose, 10g; wheat bran, 10g; soybean flour, 10g; KH_2_PO_4_, 1g and four glass beads (5 mm in diameter) to smash the pelleted mycelium. After six days of incubation in a shaker at 150 rpm, 30℃, take 10mL of culture solution, dilute with water to 90mL, mixed with 10mL of 5μmol/mL emodin suspension. Then, take the mixed liquore of 1ml after 30mim, 2h, 6h, 24h, and add double labeled fluorescent 1μL, shake up and standing for 10 minutes.

The confocal microscopy imaging was acquired with a Leica TCS SP8 confocal microscopy (Germany) with adjustable white laser (470 - 700 nm) and 63X/100X oil-immersion objective lens. The fluorescence were excited at: Emodin 405nm, Mito Tracker Deep Red 644, Cell Mask Deep Red 650nm, while the emission were collected at: Emodin 500±30nm, Mito Tracker Deep Red 660±20nm, Cell Mask Deep Red 655±20nm. Quantization performed using Fiji —a "batteries-included" distribution of Image J.

# Statistical analysis

All assays were performed three times independently, with one representative experiment shown. Data were expressed as mean ± standard deviation(SD). Statistical analysis was performed using the SPSS software (SPSS Inc., Chicago, IL, USA), when P-values<0.05 were considered statistically significant.


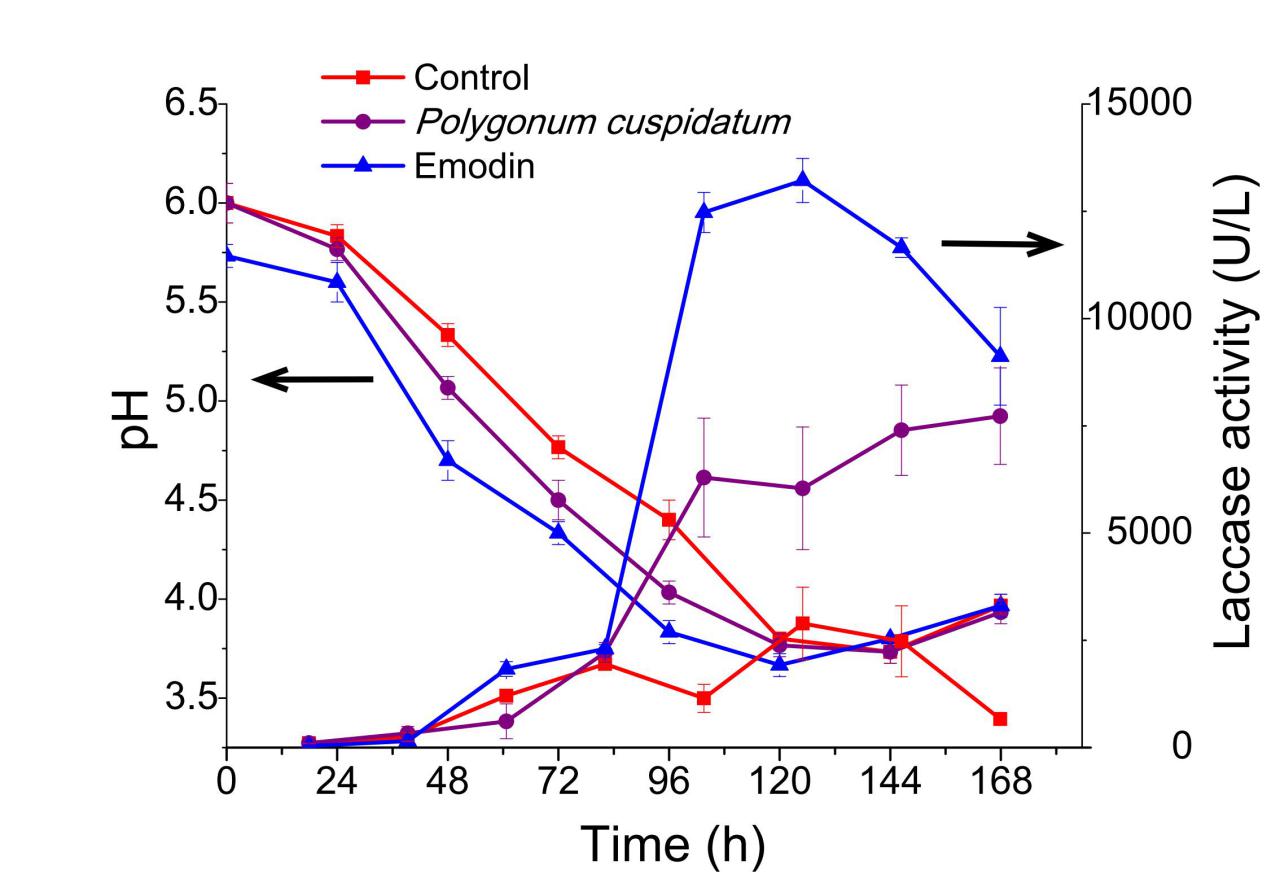


Figure S1. Induction of laccase by P. cuspidatum and emodin in 1000L fermenter filled with T. versicolor.


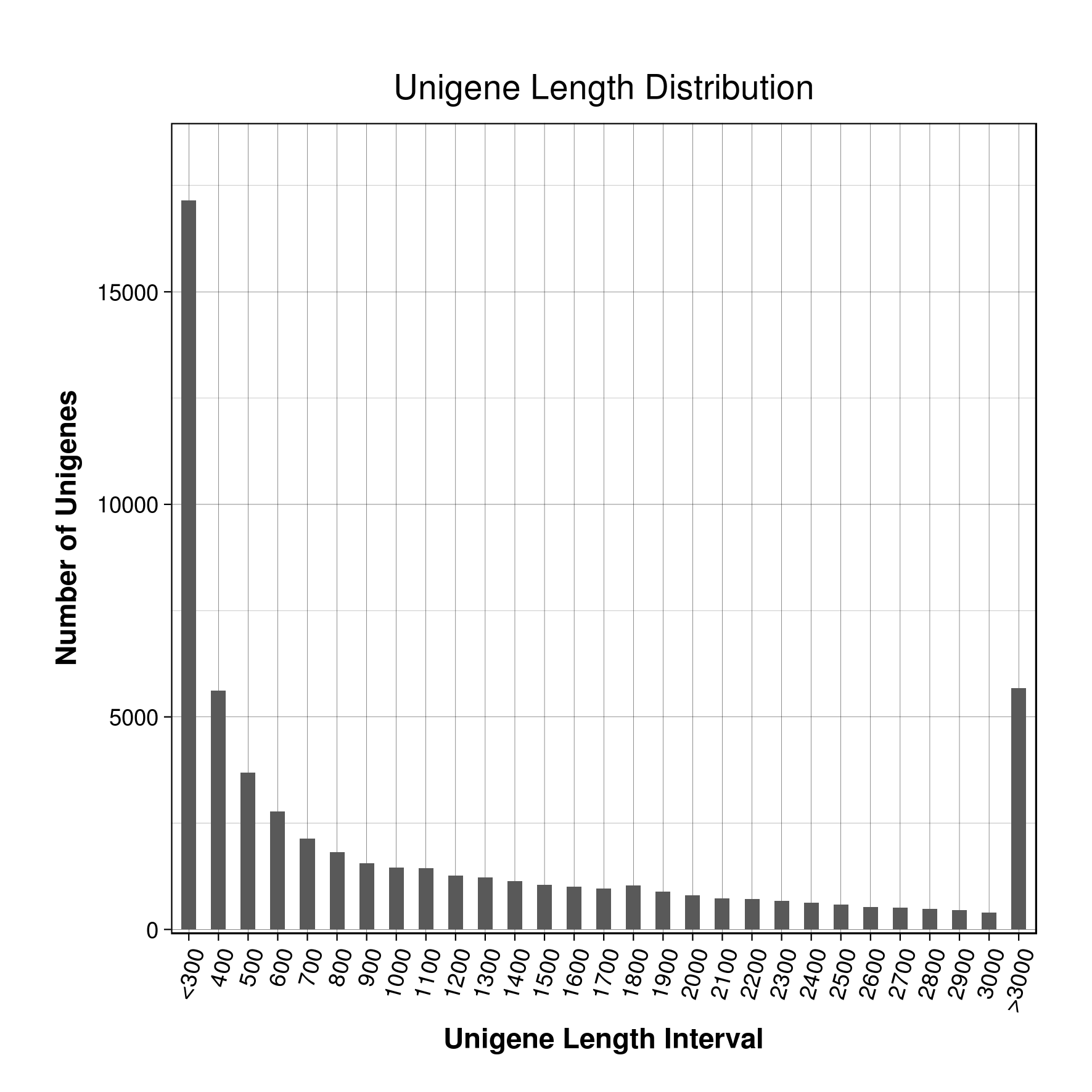


Figure S2. Unigene length distribution, the abscissa is the unigene length interval, and the ordinate is the number of occurrences of unigene for each length


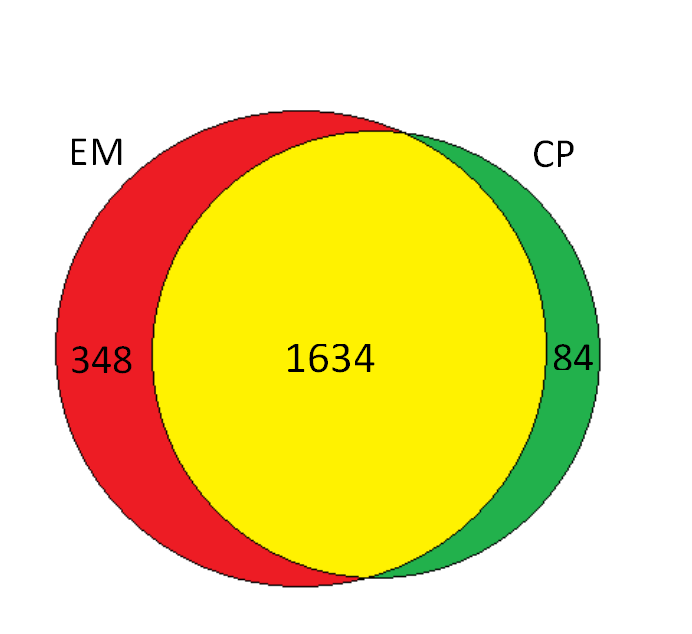


Figure S3. Areaproportional Venn diagrams depict the overlap of the identified proteins between the CK and EM bacterial strain from label-free measurements


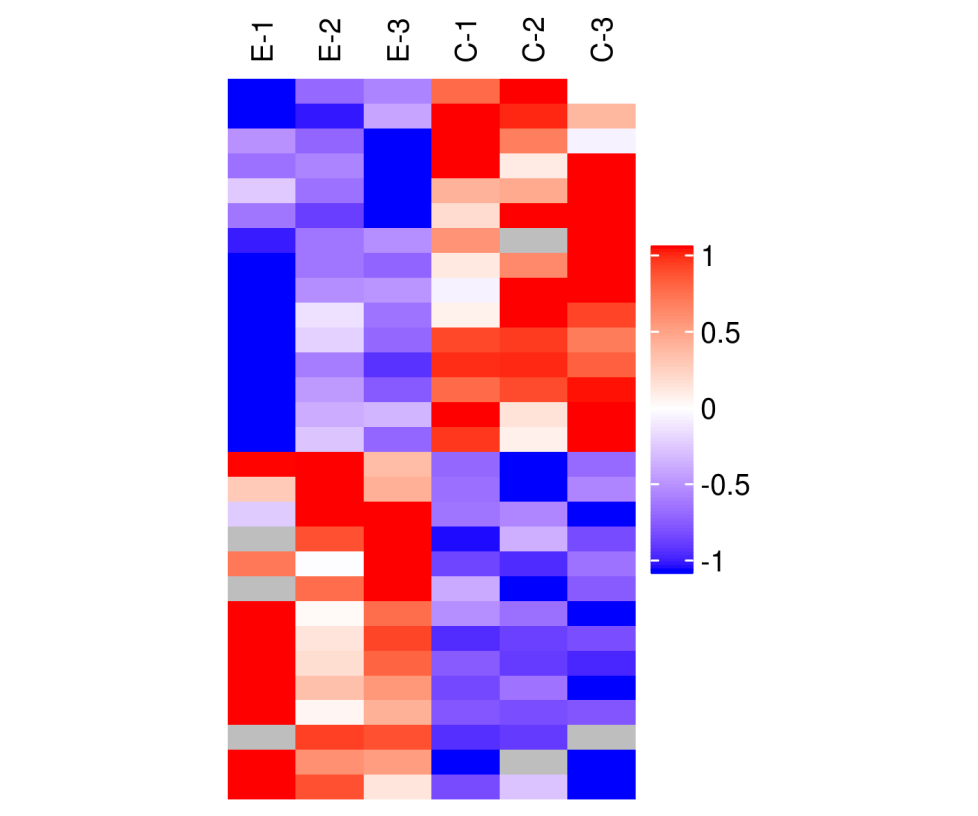


Figure S4. A heat map analysis of the CK and EM differential protein expression profiles from three replicates. I ,Blue indicates proteins that were significantly downregulated in the EM. II ,Red indicates significantly upregulated proteins in the EM


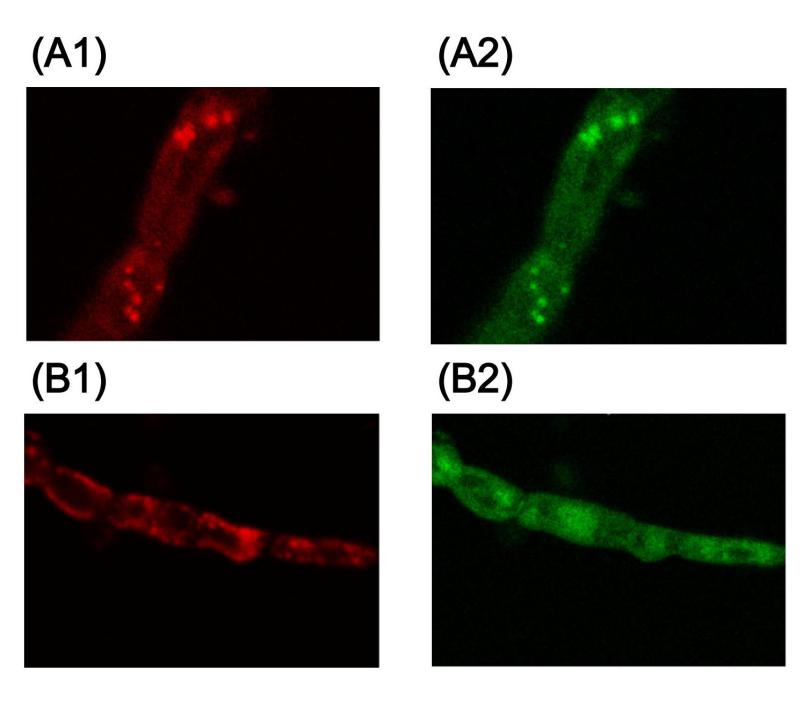


Figure S5. The confocal images of *T. versicolor* mycelium infected with Emodin shot by Confocal laser scanning microscope. The image display the location of mitochondriais (A1) and emodin (A2) after 30 minutes of incubation, and position of mitochondriais (B1) and emodin (B2) after 24 hours. Emodin has autofluorescence, the concentration in mycelium suspension is 0.5μmol/mL, and mitochondria are stained with Mito Tracker Deep Red for 1μL/mL.
